# POST DISCHARGE WALKING ACTIVITY AND 30-DAY READMISSION IN OLDER ADULTS

**DOI:** 10.1093/geroni/igz038.3304

**Published:** 2019-11-08

**Authors:** Noah Kemp, Rachel R Deer, Elena Volpi, Steve Fisher

**Affiliations:** UTMB, Galveston, Texas, United States

## Abstract

The Centers for Medicare and Medicaid Services has determined high rates of unplanned 30-day readmission to be an indicator of substandard care. More research is needed to identify strong, objective markers of readmission risk. The purpose of this analysis was to investigate the utility of average steps per day as a biomarker in determining the 30-day readmission risk of recently discharged older adults. 133 men and women, aged 65 and older, who were capable of walking on their own, recently hospitalized with an acute illness, and discharged to home were given a StepWatch Activity Monitor and monitored for up to 30 days following discharge. Average steps per day and clinical characteristics were assessed and compared with 30-day readmission. 20 of 133 participants were readmitted within 30 days. Those who were readmitted took significantly fewer steps per day overall: 4412 vs. 5948, p=0.027, and had significantly longer stays in the hospital: 4.50 vs. 2.90 days, p=0.002. Survival analysis of our sample, grouped by tertile of mean daily steps, while not statistically significant, p=0.093, demonstrated a trend toward greater probability of readmission over the 30 days post discharge for those who were in the lowest tertile. Walking activity appears to be a moderate predictor of readmission risk. A more extensive study must be conducted to better understand the relationship of walking activity after discharge and readmission.

